# Passive smoking as a risk factor of anemia in young children aged 0–35 months in Jordan

**DOI:** 10.1186/1471-2431-7-16

**Published:** 2007-04-10

**Authors:** Rathavuth Hong, Jose A Betancourt, Martin Ruiz-Beltran

**Affiliations:** 1 Department of Global Health, School of Public Health and Health Services, the George Washington University, Washington District of Columbia, USA; 2 Academy of Health Sciences, United States Army Medical Department Center & School, Fort Sam Houston, Texas, USA; 3 Southwest Washington Medical Center, 400 N.E. Mother Joseph Place, Vancouver, WA 98664, USA

## Abstract

**Background:**

Passive smoking unfavorably affects pregnancy, child birth and child health. Passive smoking associates with still-birth, premature birth as well as acute respiratory infection, asthma, disorder in red blood cell metabolism in children. This study examined the effects of passive smoking on anemia in young children in Jordan.

**Methods:**

The analysis based on the information from 740 children aged 0–35 months that were tested for hemoglobin levels included in the 2002 Jordan Population and Family Health Survey. This study used multivariate logistic regression method to analyze the effect of passive smoking on anemia in young children in Jordan, controlling for a number of risk factors and confounding factors for anemia.

**Results:**

Results indicated that independent of other risk factors and confounding factors, anemia in young children was strongly positively associated with exposure to passive smoking from both parents (OR= 2.99, *p *< 0.01). Severely undernourished children were at higher risk of anemia independent of passive smoking and other risk factors (OR= 5.29, *p *< 0.05). Children age 24–35 months, children born to mothers age 35–49, and children lived in households with a hygienic toilet facility were less likely to suffer from anemia.

**Conclusion:**

Passive smoking from both parents was strongly positively associated with anemia in young children in Jordan independent of other risk factors and confounding factors. The results support the importance of smoking prevention during and after pregnancy that prevent childhood anemia and others morbidities in young children.

## Background

Passive and active tobacco smoking exerts an extremely unfavorable effect on the course and outcome of pregnancy as well as on lactation in women [1]. Children living in the household with one or more tobacco-smokers experienced disorders of iron metabolism, hemoglobin formation, red blood cell metabolism, which led to the development of anemia during the early period of life [2,3]. Passively and actively inhaling tobacco smoke increases the blood level of carboxy-hemoglobin: a type of hemoglobin intoxicated by carbon monoxide as a result of inhaled tobacco smoke. This carboxy-hemoglobin does not have the capacity to carry oxygen to tissues and organs. A slower rate of the replacement of fetal hemoglobin or carboxy-hemoglobin by normal hemoglobin is also a pathogenesis of red blood cell- impairment in children exposed to tobacco smoke [4,5]. Passive tobacco smoking exposes young children to several toxic substances of tobacco related alkaloids. Two of these alkaloids, N-nitrosonornicotine and 4 (methylnitrosamino) -1-(3-pyridyl)-1-butanone, are strong carcinogens and found to be the causative factors in tobacco-related human lung cancer [6,7]. Passive smoking and other environmental tobacco smoking are also associated with a high prevalence of still birth, low birth weight, sudden infant death syndrome, leukemia, and acute respiratory infection and asthma in young children [8-13].

Young children are particularly at higher risk of exposure to passive smoking. Children aged less than three years olds are more likely to stay indoors almost all the time with their parent and particularly so with their mothers. This makes younger children at higher risk of inhaling passive smoking than older children who spend more time playing outdoors or going to school [14,15].

Childhood anemia is also associated with breastfeeding and feeding practices, micronutrient and nutritional intake, and other infections such as diarrhea, acute respiratory infections, parasitic infection, and malaria [16-19]. Mothers' characteristics such as age at childbirth, anemia, nutritional status, and education; and household characteristics such as household economic status, safe source of drinking water and sanitation, urban/rural residence and geographic region affect childhood anemia mediating through feeding habit, food availability, and diseases morbidities [20,21].

Using data from the 2002 Jordan Population and Family Health Survey, this study examines the relationship between childhood anemia and passive smoking from one or both parents in young children age 0–35 months.

## Methods

The analysis used data from the 2002 Jordan Population and Family Health Survey (JPFHS). The sample procedure based on a sampling frame from the 1994 Census of Population and Housing and was stratified by governorate, major cities, other urban, and rural within each stratum. The sample was selected in a two stage process. First, blocks were selected systematically as primary sampling units (PSUs) with probability proportional to size of the PSU. A total of 498 PSUs were selected at this stage. In the second stage, a fixed number of 16 households were selected in each selected PSU. The JPFHS collected demographic, socioeconomic, and health data from a nationally representative sample of 5,630 children aged 0–59 months and 5,751 women aged 15–49 in a sample of 5,590 households. The survey also measured hemoglobin levels in 27% of the children and 30% of the women that responded to the interview. Only 76% of eligible children's mothers gave consent for hemoglobin testing. The analysis included children aged 0–35 months with valid measurement of the hemoglobin level and the sample size was limited to 740 weighted numbers of children. The sampling design allowed estimations of population and health indicators at national, urban rural residence, and major geographical region levels. The non-response was not different by background characteristics for both children and women and had not caused any bias in the data. Hemoglobin testing was the primary method of anemia diagnosis. The survey included direct measurement of hemoglobin levels. Hemoglobin measurements were taken in the field using the HemoCue system (HemoCue AB, Sweden). A drop of capillary blood was taken from the finger (or from the heel in the case of infants under 6 months) and drawn in one continuous process directly into a reagent-coated microcuvette and the filled microcuvette was inserted into a cuvette holder of a portable, battery-operated photometer. Hemoglobin concentration was indicated on a digital readout in grams per deciliter. [22].

Each woman interviewed in the survey was asked if she or her husband currently smoke cigarettes or nargila. Nargila is a form of tobacco smoke that uses water pipes, which are popular in the Middle Eastern region [23]. Passive smoking was defined as children exposed to parental smoking (none of the parents, one of the parents, both of the parents). Anemia in young children and mothers was defined according to their level of hemoglobin. The measurement of hemoglobin was adjusted for the altitude of the location of the household because of a relatively higher level of hemoglobin being associated with higher altitudes due to lower concentrations of oxygen. Women and children with hemoglobin level <10.0 g/dl were moderate/severe anemic. Pregnant women with hemoglobin level 10.0–10.9 g/dl; and non-pregnant women and children with hemoglobin level 10.0–11.9 g/dl were mildly anemic. The anemia level was analyzed as not anemia/mild anemia (hemoglobin ≥ 10.0 g/dl) and moderate anemia/severe anemia (hemoglobin < 10.0 g/dl) [24].

Because anemia is correlated with a child's breastfeeding status, micronutrient supplement, child's nutritional status, maternal anemia and nutritional status, household living condition; and other child, mother, and household characteristics and socioeconomic factors, the association of passive smoking and childhood anemia was estimated after adjusting for the effects of these risk factors and confounding factors and characteristics. These factors include a child's age (0–11, 12–23, 24–35 months), sex (boy, girl), birth order (1, 2, 3, 4+), birth weight (≥ 2,500, < 2,500 g), duration of breastfeeding (never breastfed, 0–11, 12–17, 18–23, 24+ months), nutritional status (not stunted, moderately stunted, severely stunted), iron supplement intake during pregnancy (none, 1–2 trimesters, 3 trimesters); mother's anemia (no/mild, moderate/severe), age at childbirth (13–24, 25–34, 35–49 years), mother's body mass index (BMI) (18.5–24.9, <18.5, 25.0+ kg/m^2^), mother's education (no education, primary or less, secondary or higher); household wealth status (low, middle, high), access to safe drinking water (no, yes), availability of a hygienic toilet (no, yes), residence (urban, rural), and geographic region (Central, North, South). For detail definition of variables, see Table 1.

The effects of passive smoking and other factors on childhood anemia were estimated using multivariate logistic regression method in STATA [25]. The analysis was done in four consecutive models. The first model presented unadjusted effect of passive smoking on childhood anemia. In the second and third models the analysis added child characteristics and mother characteristics; while in the fourth and full models the household conditions, residential and geographical variables were additionally controlled for. In our analysis, weights were used to restore representativeness of the sample that was over-sampled and to adjust for non-response rates that varied from one geographical region to another [22]. An alternative model was used to estimate the effects of passive smoking from either mother or father on the risk of childhood anemia. Results are presented as odd ratio (OR) with statistical significant level (*p*-value).

### Ethics

This study is based on secondary analysis of existing survey data with all identifying information removed. The survey acquired informed consent from mothers of the children included in this study before asking any questions and before obtaining biologic and anthropometric measurements.

## Results

Table 1 illustrates the sample distribution of children aged 0–35 months by their passive smoking status and other selected background characteristics and risk factors. About 57% of the children were exposed to passive smoking from one of their parents and 8% to both of their parents. Forty-one percent of children were 0–11 months, 35 percent were 12–23 months, and 25 percent were 24–35 months. There were slightly more boys (53%) in the sample than girls (47%). Seventeen percent of children were first and second-order births, 20% were third-order births and 46% were fourth or higher order births. Four percent of the children had never been breastfed, 56% were breastfed less than 12 months, 24% were breastfed for 12–17 months, 12% for 18–23 months, and 5% were breastfed for more than 24 months. Only 7% of the children were chronically malnourished. About one in four children's mothers did not take iron tablets or syrup during pregnancy of the child, 59% children's mothers only took an iron supplement up to the second trimester, and 14% of them took supplement up to the third trimester. Slightly more than one in every four of the children (26%) were born to mothers aged 13–24, about 58% to mothers aged 25–34, and the remaining 17% to mothers aged 35–49. Only two percent of the children were born to undernourished mothers (BMI < 18.5 kg/m^2^), 4% were born to illiterate mothers, 9% were born to mothers with primary education or less. About 34% of the children lived in households of low wealth status and 25% in households of higher wealth status. Only 7% of the children lived in households without safe sources of drinking water and 13% lived in households without a hygienic toilet facility. Twenty-six percent of children live in rural areas. By geographic region, 66% of children lived in the Central Region, 24% lived in the Northern Region, and 10% lived in the Southern Region.

On average, 19% of the children less than three years old were anemic. The percent of anemic children was higher among children exposed to passive smoking from both parents (42%) compared to the children who were not exposed (19%), and those who were exposed to passive smoking from only one of their parents (17%). Young children were more likely to suffer from anemia than older children and the percentage of boys and girls with anemia are about the same. The percentage of anemic children varies slightly by child's birth order. Low-birth-weight children are more likely anemic than other children. Children that received some breastfeeding were less likely to suffer from anemia, particular so among those who were breastfed for 24 months or longer. Nineteen percent of well-nourished children, 21% of moderately undernourished children and 60% of severely undernourished children were anemic. Children born to mother who took iron supplement up to the third trimester of pregnancy and mothers aged 25–49 years old are less likely to be anemic. The percentage of childhood anemia was higher among children of undernourished mothers (39%) than for children of overweight mothers (19%) and of mothers with normal BMI (20%). Children of mothers with no education had a higher rate of anemia (27%) compared to children of mothers with a primary education or less (17%) and mothers with a secondary education or higher (19%).

Twenty-three percent of children who live the households of lowest wealth status were anemic compared to 17% of those in the households of middle wealth status and 19% of those in the households of highest wealth status. Lack of safe drinking water and a hygienic toilet facility were also associated with a higher prevalence of childhood anemia. As expected, children living in the rural Jordan have a higher prevalence of anemia than those living in the urban areas. The prevalence of moderate and severe anemia did not vary very much by geographic region.

### Association of passive smoking and anemia in young children

For children aged 0–35 months, results from bivariate analysis show that there is a strong relationship between childhood anemia and passive smoking from both parents, as children exposed to passive smoking from both parents are three times as likely to suffer from anemia as children that were not exposed (OR = 3.08, *p *< 0.01). However, there is no relationship between childhood anemia and passive smoking from one of parents (Table 2, Model 1). The association of passive smoking from both parents on anemia in young children remains large and highly statistically significant, when the effects of child's age, sex, birth order, birth weight, breastfeeding and nutritional status were controlled for in model 2 (OR = 3.14, *p *< 0.01). Additionally controlling for mother's iron supplement intake, age at childbirth, BMI, education (Model 3), does not affect the relation between passive smoking and anemia in young children. With all child, maternal, household, and regional factors controlled, young children who were exposed to passive smoking from both of parents remained significantly likely to be anemic (OR = 2.99, *p *< 0.01) (Model 4).

Table 3 shows the main effect of passives smoking from only mother or only father on childhood anemia. Exposure to only mother or only father is not significantly associated with childhood anemia. Only combined exposure to passive smoking has significant both unadjusted and adjusted effects of anemia in the children.

### Effects of other risk factors on childhood anemia

In model 4 in Table 2, with passive smoking exposure and other factors controlled, severely undernourished children were significantly positively associated with severe anemia (OR = 5.29, *p *< 0.05). Furthermore, older children, children of older mothers, and children living in households with hygienic toilet facilities are significantly less likely to suffer from anemia. The lack of statistically significant relationships between maternal nutritional status and anemia in children may be due to the small sample size of mothers with a BMI < 18.5 kg/m^2 ^(2%). None of the other risk factors or background characteristics had a statistically significant association with the risk of anemia in young children in Jordan.

## Discussions and conclusion

Passive smoking from both parents is significantly associated with anemia in young children. Results from this study show that anemia in children aged 0–35 months is strongly positively associated with passive smoking from both parents independent of child's age, sex, birth order, breastfeeding, nutritional status; maternal age at child birth, education and nutritional status; and household wealth status, safe sources of drinking water, hygienic sanitation, and other factors. This finding supports the evidence of the negative effects of passive smoking on health, particularly on childhood anemia. Tobacco smoke by parents adversely affects children as early as during the prenatal period through passive and/or active smoking mothers resulting in still births and premature births [26,27]. Newborns and young children passively inhaling tobacco smoke experienced disorders of iron, hemoglobin, and red blood cell metabolism, leading to the early development of anemia [28]. Passive smoking also slows the rate of the replacement of fetal hemoglobin or carboxy-hemoglobin by normal hemoglobin causing red blood cell impairment and anemia [29], and exposes young children to several carcinogenic substances of tobacco that cause human lung cancer [30]. Passive smoking and other environment tobacco smoking are also associated with a high prevalence of acute respiratory infection and asthma in young children [31].

Consistent with previous research, this study finds that maternal nutritional status has a strong association with childhood anemia [32]. However this association is not statistically significant and this may be due to the sample size. The relationship between mother's nutritional status and childhood anemia operates mainly through the dietary intake and nutritional status of mothers, and the quantity and quality of breast milk [33]. Contrary to previous studies, mother's iron intake, anemia status, education and safe sources of drinking water have no effect on the risk of anemia in young children [34,35].

This study can be criticized for a number of limitations. The first potential limitation of the study is its inability to analyze for the dose of passive smoking. Even though the analysis included passive smoke from one of the parents and from both of the parents, this information could not quantify the specific dose of exposure. The information on when the parents first began smoking, how many cigarettes or nargila that each parent smoked per day at home and indoor, how long do children spend time indoors with parent(s), and whether any other household members smoked were not collected in JPFHS. The second potential limitation of our study is that it did not account for highly polluting cooking fuels. However, almost all the households in Jordan (99%) use low polluting fuels such as electricity, natural gas, and kerosene. Therefore controlling for cooking fuel would not be necessary. This study can be criticized for its inability to control for current parasitic infections such as hookworm and malaria that can cause anemia. Nonetheless, information related to these morbidities was not available from our survey data. Also one can argue that ETS in children increases Hb levels as a consequence of an increased carboxyhemoglobin (CoHb) from inhaling carbon monoxide from ETS. Since CoHb has no oxygen carrying capacity, its presence causes a generalized increase of the Hb level similar to those observed in adults. In fact a study in Japan among adult men found an increase in hemoglobin level among smokers. Further study is needed to overcome this by conducting the analysis on the level of normal Hb and CoHb separately [36,37].

Despite these potential limitations, consistency in the strength and direction of the relationship between passive smoking and childhood anemia emphasizes the importance of smoking prevention during and after pregnancy in both parents, which could reduce many complications in children's health with a high medical, social and economical cost.

## Competing interests

The author(s) declare that they have no competing interests.

## Authors' contributions

RH carried out the study design, data management and analysis; and drafted and revised the manuscript. JAB participated in the designing of the study, and in drafting and revising the manuscript. MRB participated in the designing of the study, and in drafting and revising the manuscript. All authors read and approved the final manuscript.

## Pre-publication history

The pre-publication history for this paper can be accessed here:



## Figures and Tables

**Table 1:** Sample distribution and prevalence of anemia among children aged 0–35 months by passive smoking status and other selected characteristics, Jordan 2002

**Characteristic**	**Number of children**	**Percent distribution of children**	**Percentage of children with anemia **
**Jordan **	740	-	19.3
			
**Passive smoking status-1**			
None of parents smoke	259	35.0	18.8
One of parents smoke	423	57.1	16.5
Both parents smoke	59	7.9	41.6
**Passive smoking status-2**			
None of parents smoke	259	35.0	18.8
Mother smoke	68	9.1	37.8
Father smoke	414	55.9	16.6
**Child's age (months)**			
0–11	301	40.7	22.8
12–23	257	34.8	20.9
24–35	181	24.5	11.3
**Child's sex**			
Boy	392	52.9	18.7
Girl	348	47.1	20.0
**Child's birth order**			
1	124	16.8	21.7
2	123	16.6	20.3
3	150	20.3	17.7
4+	343	46.3	18.8
**Birth weight**			
≥ 2,500 g	665	89.9	18.2
< 2,500 g	73	9.9	27.8
**Breastfeeding status (months)**			
Never	26	3.5	24.2
0–11	415	56.1	20.3
12–17	177	24.0	17.4
18–23	88	11.9	22.1
≥ 24	34	4.6	6.2
**Stunted^†^**			
Not stunted	659	89.1	18.8
Moderately stunted	41	5.6	20.9
Severely stunted	10	1.4	60.4
**Iron intake during pregnancy**			
Not taken	199	26.9	17.3
1–2 trimesters	437	59.1	21.6
3 trimesters	100	13.5	13.8
**Mother's anemia**			
No/mild	519	70.1	17.9
Moderate/severe	213	28.2	22.8
**Mother's age at childbirth (years)**			
13–24	189	25.6	25.1
25–34	426	57.6	18.6
35–49	124	16.8	12.8
**Mother's BMI (kg/m**^2^)			
18.5 – 24.9	264	35.6	19.5
< 18.5	17	2.3	39.4
≥ 25.0	459	62.0	18.5
**Mother's education**			
No education	26	3.5	26.8
Primary or less	67	9.1	16.6
Secondary or higher	647	87.5	19.3
**Household wealth status**^¶^			
Low	250	33.8	22.5
Middle	304	41.2	17.1
High	186	25.1	18.6
**Safe drinking water^$^**			
No	54	7.3	22.1
Yes	686	92.7	19.1
**Hygienic toilet**^@^			
No	97	13.1	35.9
Yes	643	86.9	16.8
**Urban/rural**			
Urban	547	73.9	18.1
Rural	193	26.1	22.6
Geographic region*			
Central	491	66.4	19.9
Northern	175	23.7	18.2
Southern	74	10.0	17.8

^†^A stunted (chronically under-nourished) child was defined as having his/her height-for-age more than two standard deviations below the median of an international reference population recommended by the World Health Organization. Children with a height-for-age z-score below -2 standard deviations but above -3 standard deviations were defined as moderately stunted, and those below -3 standard deviations were defined as severely stunted.

^¶^Household wealth is calculated base on the possession of household assets and characteristics included radio, television, refrigerator, car, truck, main floor material, and electricity using principle component analysis.

^$^Safe sources of drinking water include piped water and tube well.

^@^Hygienic toilet includes flush (own/shared) toilet connecting to sewage or having a septic tank *Jordan is divided into 12 administrative governorates, which are grouped into three regions – the North region (Irbid, Jarash, Ajloun, and Mafraq), the Central region (Amman, Zarqa, Balqa, and Madaba), and the South region (Karak, Tafielah, Ma'an, and Aqaba).

**Table 2:** Odds ratio (OR) estimates of effects of passive smoking status and other selected characteristics on anemia among children aged 0–35 months, Jordan 2002

	**Odd Ratio (Significant Levels)**			
**Characteristic**	**Model 1**	**Model 2**	**Model 3**	**Model 4**
**Passive smoking status**				
None of parents smoke^†^	1.00	1.00	1.00	1.00
One of parents smoke	0.86	0.84	0.87	0.83
Both parents smoke	3.08***	3.14***	3.00***	2.99***
**Child's age (months)**				
0–11^†^		1.00	1.00	1.00
12–23		0.61	0.64	0.63
24–35		0.34***	0.37**	0.39**
**Child's sex**				
Boy^†^		1.00	1.00	1.00
Girl		1.01	0.99	1.01
**Child's birth order**				
1^†^		1.00	1.00	1.00
2		1.00	1.19	1.19
3		0.90	1.13	1.17
4+		0.88	1.49	1.45
**Birth weight**				
≥ 2,500 g^†^		1.00	1.00	1.00
< 2,500 g		1.50	1.63	1.60
**Breastfeeding status (months)**				
Never^†^		1.00	1.00	1.00
0–11		0.54	0.44	0.40
12–17		0.78	0.61	0.55
18–23		1.23	0.98	0.80
≥ 24		0.39	0.28	0.23
**Stunted**				
Not stunted^†^		1.00	1.00	1.00
Moderately stunted		1.17	1.21	1.19
Severely stunted		4.87**	5.54**	5.29**
**Iron intake during pregnancy**				
No^†^			1.00	1.00
1–2 trimesters			1.37	1.36
3 trimesters			0.73	0.77
**Mother's anemia**				
No/mild^†^			1.00	1.00
Moderate/severe			1.38	1.49
**Mother's age at childbirth (years)**				
13–24^†^			1.00	1.00
25–34			0.61	0.62
35–49			0.40*	0.40*
**Mother's BMI (kg/m**^2^**)**				
18.5 – 24.9^†^			1.00	1.00
< 18.5			2.60	2.79
≥ 25.0			1.01	1.06
**Mother's education**				
No education^†^			1.00	1.00
Primary or less			0.95	1.19
Secondary or higher			1.20	1.73
**Household wealth status**				
Low^†^				1.00
Middle				0.73
High				0.98
**Safe drinking water**				
No^†^				1.00
Yes				0.87
**Hygienic toilet**				
No^†^				1.00
Yes				0.43***
**Urban/rural**				
Urban^†^				1.00
Rural				1.00
Geographic region				
Central^†^				1.00
Northern				0.92
Southern				0.79
				
**Number of children**	740	726	712	712

^†^Reference group, * *p *< 0.1, ** *p *< 0.05, *** *p *< 0.001

For variable definitions, see Table 1.

**Table 3:** Odds ratio (OR) estimates of effects of passive smoking from only mother, only father, or both of parents on anemia among children aged 0–35 months, Jordan 2002

	**Odd Ratio (Significant Levels)**	
**Characteristic**	**Unadjusted**	**Adjusted**^‡^
**Passive smoking status**		
None of parents smoke^†^	1.00	1.00
Mother smokes only	0.62	1.18
Father smokes only	0.82	0.82
Both parents only	3.08***	2.99***
		
**Number of children**	740	712

^†^Reference group, * *p *< 0.1, ** *p *< 0.05, *** *p *< 0.001

^‡^The adjusted model include all variables in Table 2, but only the main effects are shown here
